# Improving the Accuracy and Equity of Pulse Oximeters: Collaborative Recommendations

**DOI:** 10.1016/j.jacadv.2022.100118

**Published:** 2022-10-28

**Authors:** Megh Rathod, Heather J. Ross, Daniel Franklin

**Affiliations:** a Institute of Biomedical Engineering, Faculty of Applied Science and Engineering, University of Toronto, Toronto, Ontario, Canada; b Translational Biology and Engineering Program, Ted Rogers Center for Heart Research, Toronto, Ontario, Canada; c Ted Rogers Center for Heart Research, Peter Munk Cardiac Center, University Health Network, Toronto, Ontario, Canada; d Division of Cardiology, Department of Medicine, University of Toronto, Toronto, Ontario, Canada

**Keywords:** cardiovascular monitoring, melanin, oxygen saturation, patient monitoring, pulse oximetry, skin tone bias, wearable devices

Pulse oximetry was invented by biomedical engineer Dr Takuo Aoyagi in 1974 to noninvasively measure arterial blood oxygen saturation. The invention was touted as “arguably the greatest advance in patient monitoring since electrocardiography”, with its introduction coinciding with a 90% reduction in anesthesia-related fatalities.^1^ It was a revolutionary concept to detect oxygen saturation without having to draw blood—saving cost and time and reducing discomfort to the patient. Pulse oximetry has since become ubiquitous for in-patient monitoring as a key vital sign for cardiopulmonary function. With recent advances in wireless technology and wearables, pulse oximeters have extended into remote patient settings and consumer markets. The demand for at-home monitoring has further expanded during the COVID-19 pandemic prompting the proliferation of low-cost, over-the-counter pulse oximeters. While pulse oximetry provides a powerful estimate of oxygen saturation and cardiopulmonary function, limitations exist given underlying assumptions and measurement confounders. For example, recent studies have documented a clinical bias where pulse oximeters overestimate blood oxygen saturation in dark-skinned patients, resulting in false negatives for hypoxia.^2^^,^^3^ These inaccuracies have also been linked to bias in treatment.^4^^,^^5^ Within this viewpoint, we highlight important limitations in data collection, study design, and overall accuracy of many current devices which we must fully understand to mitigate bias and provide effective solutions. We then give recommendations for future oximeter studies, device functionality/validation, and consumer advocacy that aim to improve the clinical use and relevancy of pulse oximeters.

Conventional pulse oximeters use 2 wavelengths of light—one below and one above 800 nm—to estimate SpO2 based on a method termed “ratios of ratios” arising from Beer-Lambert law of optical absorption. The “ratios of ratios” model relies on empirical fitting terms that manufacturers program into each device as a “factory calibration.” These proprietary calibration terms originate through device validation studies which compare pulse oximetry estimates to gold standard blood gas analysis measurements. The factory calibration accounts for specific geometries and features of the optoelectronic components used within their device but also the variation of patients used within the validation data set. This introduces the opportunity for sources of error and limited patient sampling within the original calibration data set to persist within future measurements. While the intent of the “ratio of ratios” method is to “cancel out” any static variable within the optical path, the model's efficacy and calibration recruitment choice has implications on device accuracy for patients not represented within the calibration data set.

Technology often advances faster than regulatory agencies, and demand has driven the market for over-the-counter devices, some with questioned efficacy.^6^ The global pulse oximeter market size was valued at $2.3 billion USD in 2020, with North America accounting for almost one-half of all sales.^7^ This value is expected to expand at a compound annual growth rate of 6.4% from 2021 to 2028.^7^ Handheld devices accounted for the largest revenue share of 38.9% in 2020.^7^ Hospitals and other health care facilities dominate the pulse oximeter market and accounted for the largest revenue share of 82.0% in 2020, with the homecare segment expected to have lucrative growth.^7^ These devices are being purchased at a rapid pace, but the understanding and validation of these devices have limited regulatory agency oversight. While over-the-counter consumer-ready devices raise concerns regarding lack of regulatory approval, recent findings suggest issues extend to Food and Drug Administration (FDA)-approved clinical devices as well.

The issue of racial bias in pulse oximeter measurements, an inaccuracy specifically attributed to the variable, has recently surfaced with greater attention. Sjoding et al,^2^ in a retrospective multicenter study of 10,000 patients, found that Black patients had a 2 to 3 times greater chance of unrecognized hypoxemia by pulse oximetry, when matched with White patients and validated by invasive arterial blood gas analysis. The study also controlled for major confounders of heart failure: smoking status and diabetes.^2^ This could have significant ramifications if at-risk patients are not identified with hypoxemia—a sign of impending acute or congestive heart failure.^2^ These errors lead to a phenomenon known as “occult hypoxemia”—where arterial blood oxygen saturation is <88% despite a pulse oximetry reading >92%.^2^ However, this potential inaccuracy has been highlighted earlier by groups such as the Severinghaus Lab, who explored the effect of skin pigmentation on pulse oximetry accuracy at low oxygen saturation values.^3^ They found that common pulse oximeters (Nellcor, Novametrix, and Nonin) were overestimating arterial saturation during hypoxia (when compared to end-tidal oxygen and carbon dioxide concentrations determined by mass spectrometry to estimate breath-by-breath SaO_2_).^3^ Additional groups have recapitulated this flaw in pulse oximeters and have linked this to delayed COVID-19 treatment and delayed oxygen administration.^4^^,^^5^ Current clinical decision-making tools such as EPIC with the Rothman Index and the National Health Service’s early warning score utilize pulse oximeter data as a key variable in proprietary algorithms to predict the deterioration of a patient. However, automated systems fed unvalidated data from devices with bias may perpetuate the impact of erroneous measurements while reducing onus on the care provider.

After the 2020 research efforts by Sjoding et al,^2^ and subsequent health journalism, the FDA responded in February 2022 regarding devices’ inaccuracy in people with dark skin pigmentation.^8^ Although the FDA commented on the limitations of the study by Sjoding et al^2^ to account for other potential confounders as a retrospective study, they agreed on the need to further evaluate and understand the association between skin pigmentation and oximeter accuracy.^8^ Current 510(k) Guidance only requires “2 darkly pigmented participants or 15% of the participant pool, whichever is larger.”^8^ The FDA also clarified that devices should be considered an estimate of oxygen saturation and explicitly states a ±4% standard deviation such that “if an FDA-cleared pulse oximeter reads 90%, then the true oxygen saturation in the blood is generally between 86% and 94%” for prescription oximeters.^8^ Over-the-counter devices, which do not pass through even these low regulatory standards, may be even less accurate, but there is a lack of consolidated data.^6^ It has since been announced that a public meeting of the Medical Devices Advisory Committee will be held to discuss available evidence and revaluate current guidelines. Given the increased scrutiny of oximeters, some manufacturers have published retrospective reviews of their devices in the context of self-reported ethnic and racial classification.^9^

While the results shown in the articles of Sjoding et al^2^ and Fawzy et al^4^ indicate a systemic bias in pulse oximeters between self-reported racial groups, the reliance on self-reported racial categories as an analog for skin tone indicates that the full extent of the problem is unknown and that biases may be >2% to 3% between patients within the extremes of self-reported categories. Additionally, the Medical Information Mart for Intensive Care data set used in the study of Gottlieb et al^5^ demonstrates that some clinical pulse oximeters blanketly report SpO2 values between 90% and 100% despite patients having blood oxygen between 70% and 90%. This highlights a second, equally alarming problem in which some clinical oximeters do not match manufacturer-reported accuracy and/or FDA standards in practice. This may potentially stem from the vastly different settings used to validate devices and their real-world clinical use or deviations in manufacturer-recommended best practices. Regardless, this additional inaccuracy limits the ability to monitor patients with SpO2 below 90% and further confounds our capacity to assess efficacy across skin tone.

To fully assess the impact of melanin variation in pulse oximetry estimates and outcomes, we suggest the incorporation of purposeful recruitment and the objective quantification of skin tone within future studies (Figure 1). Purposeful sampling would ensure a greater representation of skin tones are present in device validation data sets. Doing so requires additional, yet necessary, considerations on how to acquire a diverse patient population depending on geographical region and patient accessibility. Potential strategies may include recruitment based on self-reported visual comparison to chromatic scales such as the Monk Skin Types (10 types) or the Von Luschan Chromatic Scale (36 types). The Fitzpatrick phototype classification group is also widely used to classify the skin’s propensity to burn from ultraviolet exposure for the first 4 tones. However, its validity has been questioned since it was created in 1975 and then arbitrarily added type V and VI based on ethnic origin. While color classification charts may be sufficient in recruitment of patients for their ease of use, we recommend the absolute quantification of skin tone for data analytics and device validation. Objective quantification of skin tone would allow for more robust models when studying biophotonic interactions and would not be susceptible to varied ambient lighting conditions and differences in individual visual perception. Potential ways to make an objective and quantitative measure of skin chromophores (melanin and hemoglobin being the largest constituents) can be based on diffuse reflectance spectroscopy. Diffuse reflectance spectroscopy provides reflectance spectra and allows for the quantification of melanin and hemoglobin based on their different spectral properties, allowing for objective assessments. With a quantitative measure of skin tone included into future studies, the full extent of bias can be determined, and corrective measures developed and evaluated.

**Figure 1:**
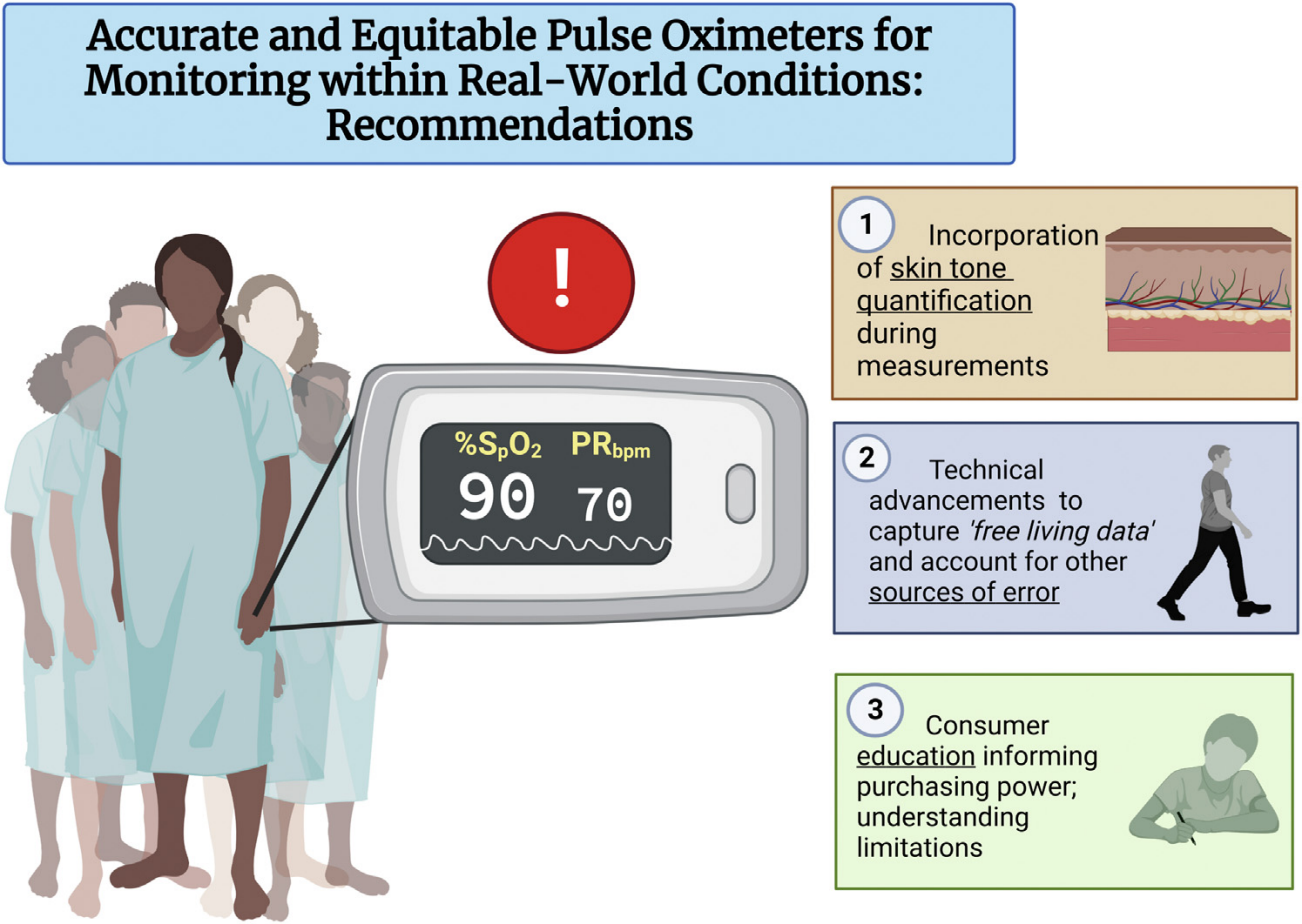
**Recommendations for More Accurate and Equitable Pulse Oximeters** 1) Incorporation of objective skin tone quantification in comparative studies to fully capture scope of bias; 2) further development of more functional devices that can accurately capture “free-living data” and account for other sources of error; 3) consumer education informing purchasing power; recognizing all devices are not created equally, and the implications these errors may have. All efforts will require a collaborative effort from various stakeholders to ensure accurate and equitable devices are achieved.

As the market for pulse oximeters and the functionality of electrical components expand, pulse oximeters must evolve to accommodate for confounding variables present in “real-world” scenarios. In its statement regarding pulse oximeters, the FDA sites multiple factors that can affect accuracy, “such as poor circulation, skin pigmentation, skin thickness, skin temperature, current tobacco use, and use of fingernail polish.”^3^ Additional sources of inaccuracy are poor perfusion, motion, excessive external light, venous pulsation, and dyshemoglobin.^1^ To address these confounders, we recommend codevelopment of oximeters with engineers, clinicians, and patients to establish devices with improved functionality, ie, temperature sensors to account for perfusion changes, additional wavelengths of light and simultaneous reflectance/transmittance measurements to account for melanin and other coloration, and pressure sensors to account for orthostatic changes, movement, and contact pressure. Validation studies with modern oximeters also need to report additional system criteria. Replicability and reproducibility, which are key in addressing the influence of skin tone on pulse oximetry, are hampered due to the lack of standardization of data collection, processing procedures, and reporting of technological factors, biobehavioral variables, and participants' demographics.^10^ These lead to limited generalizability of findings and comparison between device systems. Improved guidelines include inclusion/exclusion criteria, justification of device reliability for the study design, skin tone, body mass index, dealing with missing data, outlier identification and correction, and naturalistic use vs explicit instructions by experimenters.^10^ Lastly, we recommend bulk purchasers of pulse oximeter equipment demand more stringent validation compliance from manufacturers. Some strategies would include seeking out advanced features which accommodate for real-world sources of error and robust data sets proving efficacy below blood oxygenation of 90% and across the entire natural variation of human skin tone.

In conclusion, armed with the knowledge of these systemic issues, we have the power to push for their correction. While pulse oximetry is a powerful noninvasive estimate of arterial blood oxygen saturation and has been revolutionary, the expanding prevalence and use case of oximeters demands a more stringent look at their inaccuracies. In the best-case scenario, users are knowledgeable of underlying assumptions and accuracy of the tool. But in the worst case, the user is unaware of these biases/sources of error and may miss instances of occult hypoxemia or pass reported SpO2 values on with higher confidence than should be bestowed. To fully know the extent of this bias, we recommend purposeful recruitment and objective skin tone quantification in future studies and device validation. Lastly, we posit that sensor technology is equipped to tackle these biases and inaccuracies, but more stringent regulation and demand from consumers/hospital systems are required to incentivize their development. Through the collaboration of all stakeholders, clinical pulse oximeters can be made more accurate and equitable.

## Funding support and author disclosures

The authors have reported that they have no relationships relevant to the contents of this paper to disclose.
